# Electrocardiographic and clinical predictors for in-hospital mortality in patients with acute myocardial infarction caused by left main coronary artery occlusion

**DOI:** 10.1097/MD.0000000000043287

**Published:** 2025-07-11

**Authors:** Chunwei Liu, Fan Yang, Yuecheng Hu, Jingxia Zhang, Hongliang Cong, Ximing Li

**Affiliations:** a Department of Cardiology, Tianjin Chest Hospital, Tianjin University, Tianjin, China; b Department of Diagnostic Ultrasound, Tianjin Medical University Cancer Institute and Hospital, National Clinical Research Center of Cancer, Key Laboratory of Cancer Prevention and Therapy, Tianjin, China.

**Keywords:** electrocardiographic, in-hospital mortality, left main occlusion, shock, STEMI

## Abstract

The data on prediction for early mortality in patients with acute myocardial infarction (AMI) caused by total left main (LM) occlusion are limited. We aim to evaluate predictors for early mortality in these extremely high-risk patients. In this retrospective study, all consecutive patients with total occlusive LM-AMI were included between January 1997 and October 2023. The ECG and clinical data were compared between the in-hospital mortality and survival groups. The receiver operating characteristic curve (ROC) was created to identify the best predictors of in-hospital mortality. The primary endpoint was in-hospital mortality. Secondary outcomes included major adverse cardiovascular events. A total of 116 patients were included. The in-hospital mortality was 47% (54/116), and the 5-year MACE-free survival rate was 44%. ST-elevation myocardial infarction (STEMI), cardiogenic shock, STEMI plus shock, STEMI plus left anterior fascicular block (LAFB) + right bundle branch block (RBBB), post-procedural TIMI I to II flow, and collateral flow absence were associated with in-hospital death, with the area under ROC (AUC) of 0.726, 0.704, 0.782, 0.605, 0.633, and 0.671, respectively. ST-elevation in aVR and ST-elevation in aVR + aVL were more common in the survival group. STEMI plus shock showed a significantly greater AUC than the other predictors (82% specificity and 74% sensitivity, Z = 1.980, *P* < .05). STEMI plus LAFB + RBBB predicted in-hospital mortality with a specificity of 95% and a sensitivity of 26%. ECG features were associated with shock and collateral circulation. Patients with shock presented with more STEMI, LAFB + RBBB, STEMI plus LAFB + RBBB, collateral flow absence, prolonged QRS interval, and less ST-elevation in aVR (*P* < .05). STEMI plus shock is a valuable predictor for in-hospital mortality in patients with acute total LM occlusion. STEMI plus LAFB + RBBB predicted a fatal in-hospital outcome in LM occlusion with high specificity.

## 1. Introduction

Acute myocardial infarction (AMI) caused by acute total unprotected left main (LM) coronary artery occlusion is an uncommon clinical entity but often results in severe clinical catastrophic events, including severe circulatory failure, cardiac arrest, and sudden cardiac death. Despite advances in medical and percutaneous coronary intervention treatment during the last 2 decades, the in-hospital mortality rate of this extremely high-risk ST-elevation myocardial infarction (STEMI) subset is 54% to 56%,^[1,2]^ which is much higher than that in non-LM-AMI. Several small studies have reported that prolonged QRS interval, cardiogenic shock, out-of-hospital cardiac arrest, and collateral flow absence predicted early mortality, while post-procedural thrombolysis in myocardial infarction III flow (TIMI) in both left anterior descending coronary artery (LAD) and left circumflex artery (LCX) led to a better outcome.^[3–6]^ However, data regarding early mortality prediction is still limited due to the small sample sizes. Since most of the deaths occurred within the first few days after admission, it was essential to find simple and available preoperative markers that predict future clinical outcomes in these critically ill patients. Our previous study has reported combining electrocardiographic (ECG) criteria for predicting acute total LM occlusion as follows^[7]^: ST-elevation (STE) in aVR and aVL, STE in I, aVL, V2-V5 without V1, left anterior fascicular block (LAFB) + right bundle branch block (RBBB). In the present study, we aimed to evaluate the electrocardiographic and clinical characteristics associated with in-hospital prognosis and to identify the optimum combination of predictors in these patients.

## 2. Methods

### 2.1. Study patients

In this retrospective, single-center study, consecutive patients with LM-AMI who underwent emergency coronary angiography at Tianjin Chest Hospital, Tianjin, China, were enrolled between January 1997 and October 2023. AMI was defined according to the previously established criteria^[8]^: acute myocardial injury with detection of a rise and/or fall of cTn values with at least 1 value above the 99th percentile URL and at least one of the following: symptoms of myocardial ischemia; new ischemic ECG changes; development of pathological Q waves; imaging evidence of new loss of viable myocardium or new regional wall motion abnormality; identification of a coronary thrombus by angiography or autopsy. This retrospective analysis was performed by ethical principles consistent with the Declaration of Helsinki. The Ethics Committee of the Institute of Tianjin Chest Hospital approved the study protocol (2023LW-014). Inclusion criteria were patients presenting with AMI caused by LM culprit lesion (visible thrombus and 100% stenosis, TIMI 0). Exclusion criteria were a prior coronary artery bypass graft surgery history and no other exclusion criteria. The need for consent was waived by the ethics committee for this retrospective study.

### 2.2. Clinical and ECG characteristics

Baseline clinical characteristics and procedural and ECG data were retrieved for all selected patients. Cardiogenic shock was defined as systolic blood pressure < 90 mm Hg for at least 30 minutes or the need for supportive measures to maintain a systolic blood pressure > 90 mm Hg and signs of end-organ hypoperfusion. In case of dynamic ECG changes, we analyzed the latest ECG data before coronary angiography. RBBB was described as a QRS duration of ≥ 120 ms and a terminal R wave in lead V1; LAFB was described as a frontal plane axis between − 45° and − 90°, qR pattern in lead aVL, R-peak time in lead aVL of ≥ 45 ms, and QRS duration < 120 ms.^[9]^ STE threshold was defined as: ≥2.5 mm in men < 40 years, ≥2 mm in men ≥ 40 years, or ≥ 1.5 mm in women regardless of age in leads V2 to V3 and/or ≥ 1 mm in the other leads. Two investigators (JXZ and YCH) analyzed all digital ECGs separately, blinded to the angiographic findings, and a final decision was made after consultation with HLC in case of disagreement. According to the STE characteristics, the ECGs of these patients were categorized into 3 main patterns as previously described^[7]^: STEMI type: anterior and lateral infarction appearance with STE in leads V1/V2 to V5/V6 and in leads I and aVL; aVR type: STE in lead aVR or both leads aVR and V1, along with multiple ST-segment depressions; aVR and aVL type: STE in leads aVR + aVL or in leads I, aVL, and aVR without STE in the anterior leads.

### 2.3. Coronary angiography and clinical outcomes

Two independent cardiologists (HLC and JXZ) assessed coronary angiographic data. The collateral circulation was classified into 4 types according to different collateral filling territories as previously described^[7]^: LAD, LCX, both LAD and LCX, and no collateral circulation. Multiple experienced and competent operators performed emergency percutaneous coronary intervention. The use of antiplatelet drugs, thrombus aspiration devices, intra-aortic balloon pump (IABP), extracorporeal membrane oxygenation (ECMO), and a strategy of complete revascularization in patients with right coronary artery (RCA) stenosis was at the discretion of the operator. The primary endpoint for this analysis was in-hospital mortality. Secondary outcomes were long-term major adverse cardiac events (MACE), defined as all-cause death, stroke, and nonfatal AMI.

### 2.4. Statistical analysis

Categorical variables were presented as counts and percentages and compared using the χ^2^ test or the Fisher exact test. Continuous variables were shown as mean ± SD and compared using the Student *t*-test. The ECG parameters and clinical data were compared between the in-hospital mortality and survival groups. The Kaplan–Meier curve was performed to analyze cardiovascular event-free survival during the follow-up period. We use receiver operating characteristic curve (ROC) analysis to evaluate the prognostic significance of risk factors for in-hospital mortality. A multivariable logistic regression analysis was performed to estimate the independent predictors of in-hospital mortality with a stepwise selection of the variables exhibiting *P* < .05 in the univariate analysis. Listwise deletion methods were used for missing data. SPSS Statistics (IBM, version 26, Chicago) was used to analyze data. A 2-tailed *P*-value of < .05 was considered statistically significant.

## 3. Results

**Figure 1.**
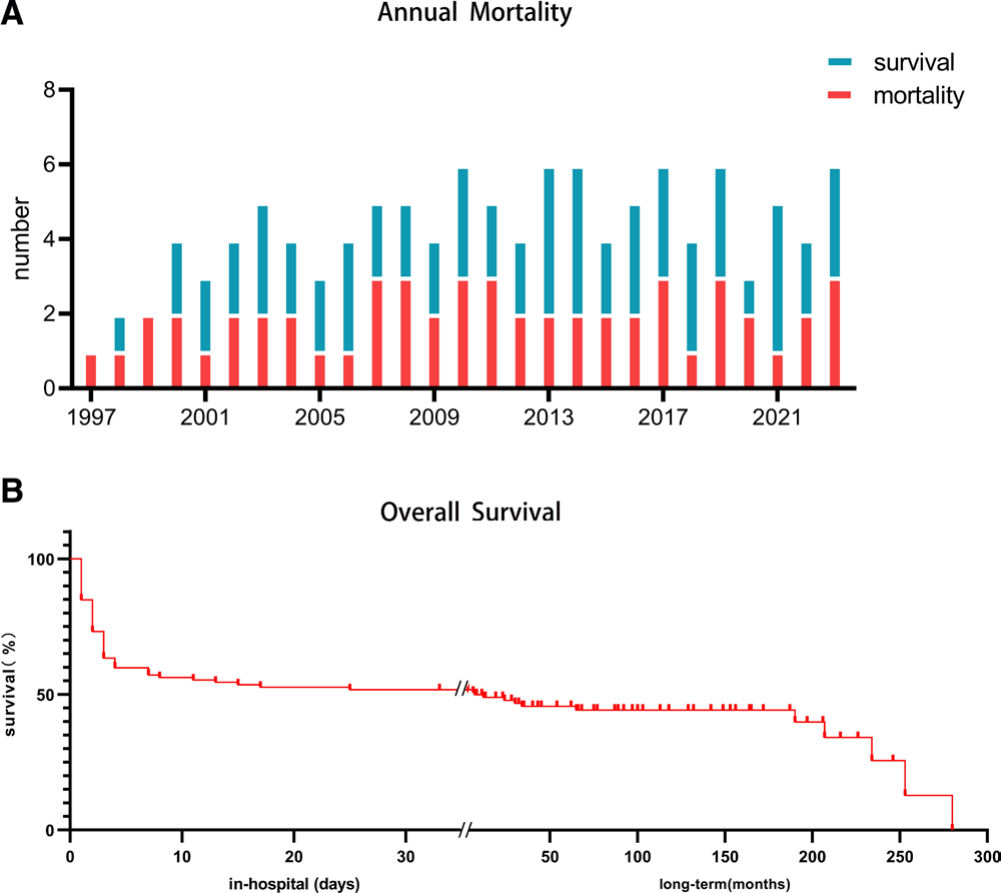
(A) The annual in-hospital mortality rate in patients with acute total occlusive LM artery from 1997 to 2023. (B) Kaplan–Meier survival curve of major adverse cardiovascular events within the median follow-up period of 100 mo. The cumulative MACE-free survival rates were 49% at 1 year and 44% at 5 years. LM = left main, MACE = major adverse cardiac events.

A total of 116 LM-AMI individuals (age range: 30–88 years, Q1: 54 and Q3: 71) were selected from 21,350 consecutive AMI patients (0.5%). The selected 116 patients comprised 96 men and 20 women with a mean age of 61 ± 13 years. Three patients underwent emergent coronary artery bypass grafts after coronary angiography. There were no significant changes in the incidence and mortality rates of acute total LM occlusion during the last 2 decades (Fig. 1A, *P* < .05). Sixty-two patients survived to discharge, whereas 54 died of LM-AMI. The in-hospital mortality was 47%, of whom 31% (17/54) died within the first 24 hours, and 76% (41/54) died within 72 hours after admission. Figure 1B shows the Kaplan–Meier curves for MACE in patients with acute LM occlusion. Patients were followed up by telephone or outpatient clinical visits from November 2023 to December 2023. The median follow-up duration was 100 months (range: 1–280, Q1: 43 and Q3: 165). Long-term follow-up was available in 58 cases among the patients surviving hospital discharge, and 4 patients lost contact. A total of 12 MACEs were reported during long-term follow-up, including 7 deaths, 3 reinfarctions, and 2 strokes. Forty-six patients had no MACE during the follow-up. Of these patients who survived hospital discharge, only 1 received an implantable cardioverter defibrillator. The cumulative MACE-free survival rates were 94% at 1 year and 86% at 5 years in the patients who survived the LM-AMI.

### 3.1. Clinical characteristics and in-hospital mortality

The clinical characteristics of the 116 patients were summarized in Table 1. A total of 80 (69%) patients presented with cardiogenic shock. The in-hospital mortality was 91% among patients presenting with cardiogenic shock and 50% among those without cardiogenic shock. A successful revascularization procedure was defined as post-procedural TIMI III flow in both LAD and LCX, and TIMI III flow was achieved in 59% of all patients. Post-procedural TIMI I to II flow was observed in 34 cases in the distal LAD and 14 cases in both LAD and LCX. IABP was implanted in 90% of all patients, while mechanical ventilation was used in 48%. Intravascular microaxial left ventricular assist devices were not available in our center. ECMO has been available in recent years and utilized in 3 patients, but only 1 survived. Provisional stenting from the LM to the LAD was the preferred strategy in 111 patients. Early complete revascularization (index procedure) was achieved in 21% (6/29) of patients with severe RCA stenosis, and delayed complete revascularization (after discharge) was achieved in 10 patients. Stent implantation was not performed in 1 case of <50% residual stenosis after thrombus aspiration. Kissing balloon inflation after LM-LAD provisional stenting was performed in 27% of all patients. The 2-stent technique was used on only 1 patient, but the patient did not survive hospital discharge.

**Table 1:** Comparison of baseline clinical data between survival and in-hospital mortality groups.

	Total patients (n = 116)	Survival group (n = 62)	Mortality group (n = 54)	*t/*χ^2^	*P*-value
Age	61.2 ± 12.7	59.0 ± 13.1	63.6 ± 11.9	1.985	.050
Male/female	96 (83%)	54 (87%)	42 (78%)	1.757	.185
Hypertension	70 (60%)	35 (56%)	35 (65%)	0.844	.358
Diabetes	37 (32%)	18 (29%)	19 (35%)	0.503	.478
Smoking	91 (78%)	48 (77%)	43 (80%)	0.083	.773
Prior stroke	9 (8)	5 (8%)	4 (7%)	0.017	.895
eGFR (mL/min 1.73 m^2^)	59 ± 22	61 ± 22	56 ± 22	−1.046	.298
LDL (mmol/L)	3.30 ± 0.84	3.37 ± 0.88	3.23 ± 0.79	-0.858	.392
Cardiogenic shock	80 (69%)	31 (50%)	49 (91%)	22.383	<.01*
Cardiac arrest	23 (20%)	10 (16%)	13 (24%)	1.146	.284
Onset time (h)	3.9 ± 2.5	3.6 ± 1.9	4.1 ± 3.0	1.179	.241
Collateral perfusion	47 (41%)	35 (57%)	12 (22%)	14.031	<.01*
IABP	104 (90%)	55 (89%)	49 (91%)	0.128	.720
Mechanical ventilation	50 (43%)	22 (36%)	28 (52%)	3.153	.076
Thrombus aspiration	34 (29%)	17 (27%)	17 (32%)	0.230	.632
Final TIMI III flow in both LAD and LCX	68 (59%)	44 (71%)	24 (44%)	8.370	.004*
RCA severe stenosis	29 (25%)	16 (26%)	13 (24%)	0.046	.830
RCA PCI during the index	6 (5%)	4 (7%)	2 (4%)	0.444	.505
RCA PCI after discharge	10 (16%)	10 (16%)	0 (0%)	–	–
Additional LAD severe stenosis	35 (30%)	15 (21%)	20 (37%)	2.260	.133
Additional LCX severe stenosis	28 (24%)	12 (19%)	16 (30%)	1.664	.197
LM site					
Ostium	0 (0%)	0 (0%)	0 (0%)	0.207	.649
Body	77 (66%)	40 (65%)	37 (69%)		
Bifurcation	39 (34%)	22 (36%)	17 (32%)		
Previous LM stent	3 (3%)	1 (2%)	2 (4%)	0.501	.479
AMI history	13 (11%)	5 (8%)	8 (15%)	1.322	.250
Stent type					
Drug-eluting stent	94 (82%)	50 (82%)	44 (82%)	0.005	.946
Bare metal stent	21 (18%)	11 (18%)	10 (19%)		
Antiplatelet drug					
Clopidogrel	65 (56%)	32 (52%)	33 (61%)	1.057	.304
Ticagrelor	51 (44%)	30 (48%)	21 (39%)	1.057	.304
GP IIb/IIIa inhibitors	42 (36%)	23 (37%)	19 (35%)	0.046	.831
WBC (10^9^/L)	14.6 ± 5.6	13.2 ± 3.8	16.2 ± 6.7	1.828	.078
Hemoglobin (g/L)	139 ± 21	139 ± 19	138 ± 24	–0.255	.806
CK (U/L)	7640 ± 4361	5820 ± 3770	10098 ± 3942	3.772	.001*
CKMB (U/L)	596 ± 360	442 ± 282	796 ± 357	3.763	.001*

AMI = acute myocardial infarction, CK = creatine kinase, CKMB = creatine kinase-MB, eGFR = estimated glomerular filtration rate, GP IIb/IIIa inhibitors = glycoprotein IIb/IIIa inhibitors, IABP = intra-aortic balloon pump, LAD = left anterior descending coronary artery, LCX = left circumflex artery, LDL = low-density lipoprotein, LM = left main, PCI = percutaneous coronary intervention, RCA = right coronary artery, TIMI III flow = thrombolysis in myocardial infarction III flow, WBC = white blood cell.

* *P* < .05.

In-hospital mortality was significantly higher among patients with cardiogenic shock, older age, collateral circulation absence, and post-procedural TIMI I to II flow in either LAD or both LAD and LCX (*P* < .05). Age, sex, time of onset, cardiac arrest, and risk factors of cardiovascular disease did not differ significantly between non-survivors and survivors. The use of thrombus aspiration devices, mechanical ventilation, IABP, drug-eluting stents, triple antiplatelet therapy, and complete revascularization did not improve clinical outcomes. Echocardiograms were not performed in 7 patients due to early death. The average left ventricular ejection fraction was 34.4 ± 4.8% in 109 patients. The available left ventricular ejection fraction was higher in the survival group compared with the mortality group (32.4 ± 4.4 vs 35.8 ± 4.6%, *P* < .01). However, left ventricular end-diastolic dimension, left atrial anteroposterior diameter, pulmonary artery pressure, and moderate to severe mitral regurgitation did not differ significantly between non-survivors and survivors (Table 2). Cardiac rupture was examined by echocardiography in 4 cases of the mortality group.

**Table 2:** Comparison of echocardiographic data between survival and in-hospital mortality groups in 109 available patients.

	Total patients (n = 109)	Survival group (n = 62)	Mortality group (n = 47)	*t/*χ^2^	*P*-value
LVEF (%)	34.4 ± 4.8	35.8 ± 4.6	32.4 ± 4.4	−3.854	<.01*
LVEDD (mm)	52.8 ± 4.7	52.7 ± 4.7	52.9 ± 4.9	0.082	.935
LAAPD (mm)	39.0 ± 4.3	39.2 ± 4.6	38.5 ± 3.9	−0.462	.647
MR	11 (10%)	4 (7%)	7 (15%)	2.100	.147
PAP (mm Hg)	31.6 ± 3.1	31.5 ± 2.8	31.8 ± 3.4	0.637	.525

LAAPD = left atrial anteroposterior diameter, LVEDD = left ventricular end-diastolic dimension, LVEF = left ventricular ejection fraction, MR = moderate to severe mitral regurgitation, PAP = pulmonary artery pressure.

* *P* < .05.

### 3.2. ECG features associated with in-hospital mortality and cardiogenic shock

The most common ECG type for acute total LM occlusion was STE in anterior and lateral leads (60, 52%), followed by STE in aVR + aVL (34, 29%) and STE in aVR (22, 19%). LAFB, RBBB, and LAFB + RBBB were present in 35, 5, and 22% of all patients. The left bundle branch block was present in only 1 patient. Three de Winter electrocardiographic pattern cases evolved to ST-elevation within hours of presentation.^[10]^ One patient with STE in aVR received coronary angiography 24 hours after admission, and well-developed collateral circulation was observed. The mean door-to-balloon time in patients with STE in aVR, STE in aVR + aVL, and STEMI patterns was 164.8 ± 274.4, 103.3 ± 30.7, and 102.6 ± 32.7 minutes, respectively. The mean door-to-balloon time did not differ significantly between the 3 different ECG types (*P* > .05). There was no significant difference in the ECG patterns and bundle branch blocks after adjusting for sex, age, risk factors of cardiovascular disease, and time of onset.

**Figure 2.**
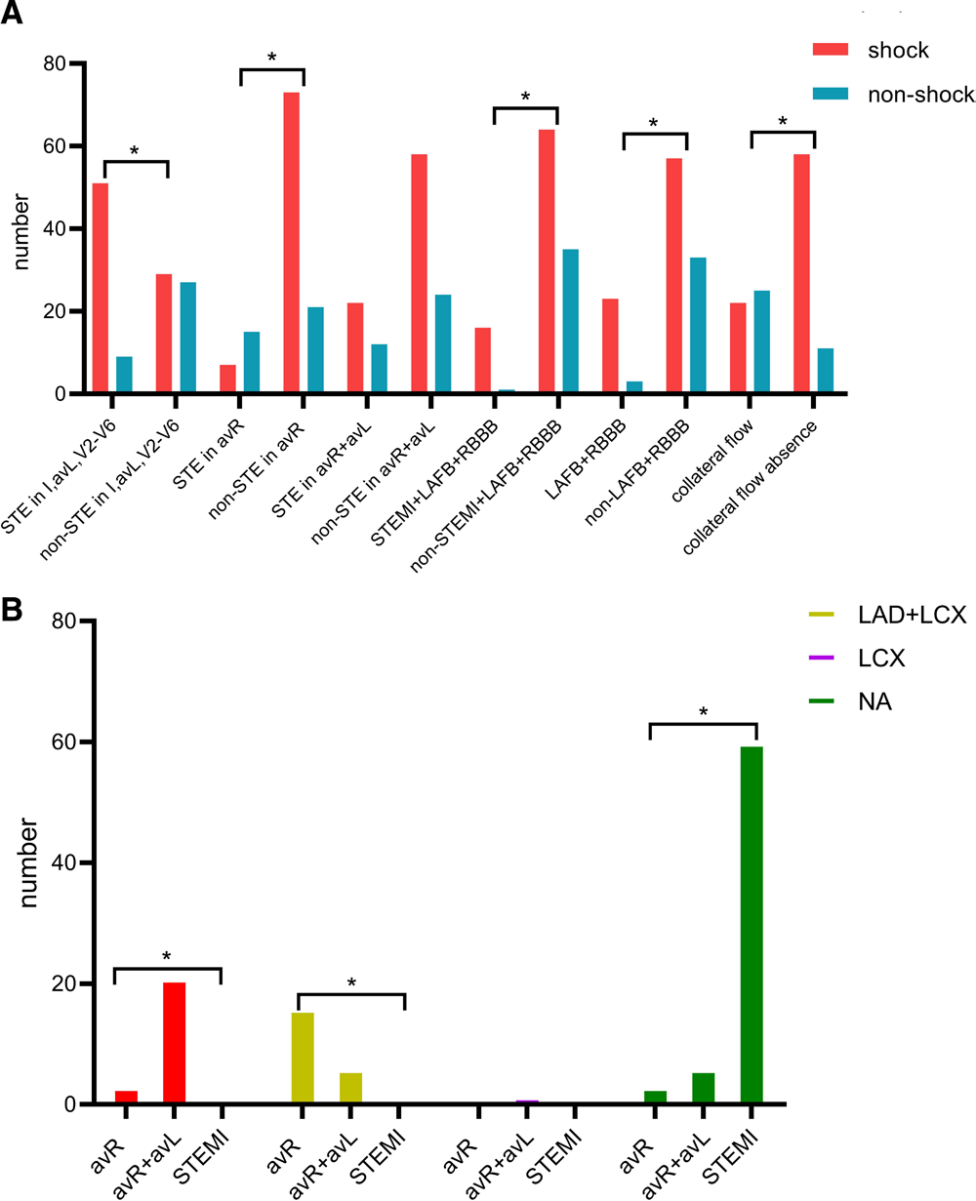
(A) The relationships between electrocardiographic features, collateral circulation, and shock in acute total LM occlusion. (B) The relationship between the electrocardiographic features and collateral circulation filling territory. *P < .05. STE = ST-segment elevation, STEMI = ST-segment elevation myocardial infarction, RBBB = right bundle branch block, LAFB = left anterior fascicular block.

STE in aVR and STE in aVR + aVL patterns were more common in patients who survived during AMI admission. In contrast, STEMI, prolonged QRS interval, and LAFB + RBBB were more common in patients who died during AMI admission (Table 3, *P* < .05). In-hospital mortality was significantly higher in STEMI patients compared with patients who met the very high-risk non-ST-elevation myocardial infarction (NSTEMI) criteria (*P* < .01). Patients with shock presented with prolonged QRS interval, more STEMI, LAFB + RBBB, STEMI plus LAFB + RBBB, collateral flow absence, and less STE in aVR (Fig. 2A, *P* < .05). The QRS interval was longer in patients with shock than those without shock (122 ± 24 vs 106 ± 27, *P* < .05). However, STE in aVR + aVL, isolated LAFB, and isolated RBBB were not associated with shock (*P* > .05).

**Table 3:** Univariate analysis of electrocardiographic predictors for in-hospital death in LM coronary artery occlusion.

	Total patients (n = 116)	Survival group (n = 62)	Mortality group (n = 54)	*t/*χ^2^	*P*-value
ECG pattern					
STE in aVR	15 (13%)	11 (18%)	4 (7%)	25.432	<.01*
STE in aVR + V1	7 (6%)	7 (11%)	0 (0%)		
STE in I, aVL, V2-V5	60 (52%)	19 (31%)	41 (76%)		
STE in aVR + aVL	34 (29%)	25 (40%)	9 (17%)		
Risk stratifies					
STEMI	60 (52%)	19 (31%)	41 (76%)	23.815	<.01*
Very high-risk NSTEMI	50 (43%)	38 (61%)	12 (22%)		
High-risk NSTEMI	6 (5%)	5 (8%)	1 (2%)		
QRS interval (ms)	117 ± 26	112 ± 25	123 ± 25	2.532	.013*
LAFB	41 (35%)	23 (37%)	18 (33%)	0.179	.672
RBBB	6 (5%)	2 (3%)	4 (7%)	1.029	.310
LAFB + RBBB	26 (22%)	8 (13%)	18 (33%)	6.927	.008*
STEMI + LAFB + RBBB	17 (15%)	3 (5%)	14 (26%)	10.261	<.01*
STEMI + shock	51 (44%)	11 (18%)	40 (74%)	37.177	<.01*

ECG = electrocardiographic, LAFB = left anterior fascicular block, LM = left main, NSTEMI = Non-ST-segment elevation myocardial infarction, RBBB = right bundle branch block, STE = ST-segment elevation, STEMI = ST-segment elevation myocardial infarction.

* *P* < .05.

### 3.3. Combining factors for the prediction of in-hospital mortality

**Figure 3.**
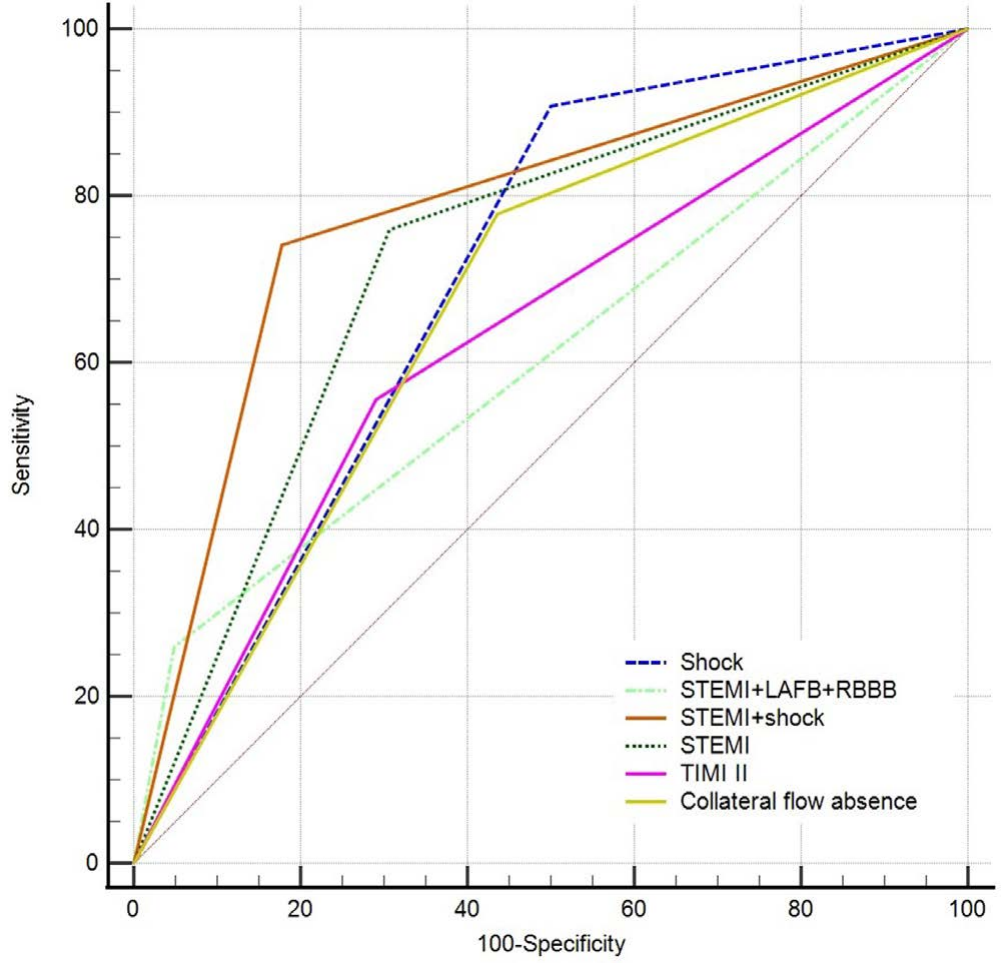
Comparison of predictors for in-hospital mortality in the ROC curve. STEMI = ST-segment elevation myocardial infarction, RBBB = right bundle branch block, LAFB = left anterior fascicular block.

ROC curves were produced to compare the predictive ability of ECG and clinical factors. The areas under the ROC curve (AUC) for STEMI, cardiogenic shock, STEMI plus shock, STEMI plus LAFB + RBBB, post-procedural TIMI I to II flow, and collateral flow absence were 0.726, 0.704, 0.782, 0.605, 0.633, and 0.671, respectively (Fig. 3). The DeLong test indicated that STEMI plus shock showed a significantly greater AUC than the other predictors, with a specificity of 82% and a sensitivity of 74% (Z = 1.980, *P* < .05). Moreover, our study demonstrated that STEMI plus LAFB + RBBB predicted in-hospital death with a specificity of 95% and a sensitivity of 26% (Table 4). Variables with a *P*-value < .05 in the univariate analysis were entered into the multivariable logistic regression. By multivariable logistic regression analysis, STEMI plus shock and post-procedural TIMI I to II flow were identified as independent risk factors for in-hospital mortality (Table 5). In subsets of patients presenting with high-risk factors (STEMI plus shock or STEMI plus LAFB + RBBB), the use of thrombus aspiration devices, mechanical ventilation, IABP, kissing balloon inflation, and triple antiplatelet therapy did not show clinical benefits (*P* > .05).

**Table 4:** The predictive ability of different electrocardiographic and clinical factors for in-hospital mortality.

Variable	Sensitivity (%)	Specificity (%)	AUC
Shock	90.7	50.0	0.704
STEMI + LAFB + RBBB	25.9	95.2	0.605
STEMI + shock	74.1	82.3	0.782
STEMI	75.9	69.4	0.726
TIMI II	55.6	71.0	0.633
Collateral flow absence	56.5	77.8	0.671

Post-procedural thrombolysis in myocardial infarction I to II flow in either left anterior descending coronary artery or both left anterior descending coronary artery and left circumflex artery.

AUC = area under the ROC curve, LAFB = left anterior fascicular block, RBBB = right bundle branch block, STEMI = ST-segment elevation myocardial infarction, TIMI II = thrombolysis in myocardial infarction II.

**Table 5:** Independent predictors of in-hospital mortality in the multivariate analysis.

Variable	Odds ratio (95% CI)	*P*-value
QRS interval	1.009 (0.990–1.028)	.373
STEMI + LAFB + RBBB	1.252 (0.249–6.288)	.785
TIMI II	2.686 (1.031–7.002)	.043*
Collateral flow absence	3.030 (0.578–15.878)	.190
STEMI plus shock	25.039 (4.689–133.698)	<.01*

TIMI II means post-procedural thrombolysis in myocardial infarction I to II flow in either left anterior descending coronary artery or both left anterior descending coronary artery and left circumflex artery.

LAFB = left anterior fascicular block, RBBB = right bundle branch block, STEMI = ST-segment elevation myocardial infarction.

* *P* < .05.

### 3.4. Relation between ECG features and collateral flow

**Figure 4.**
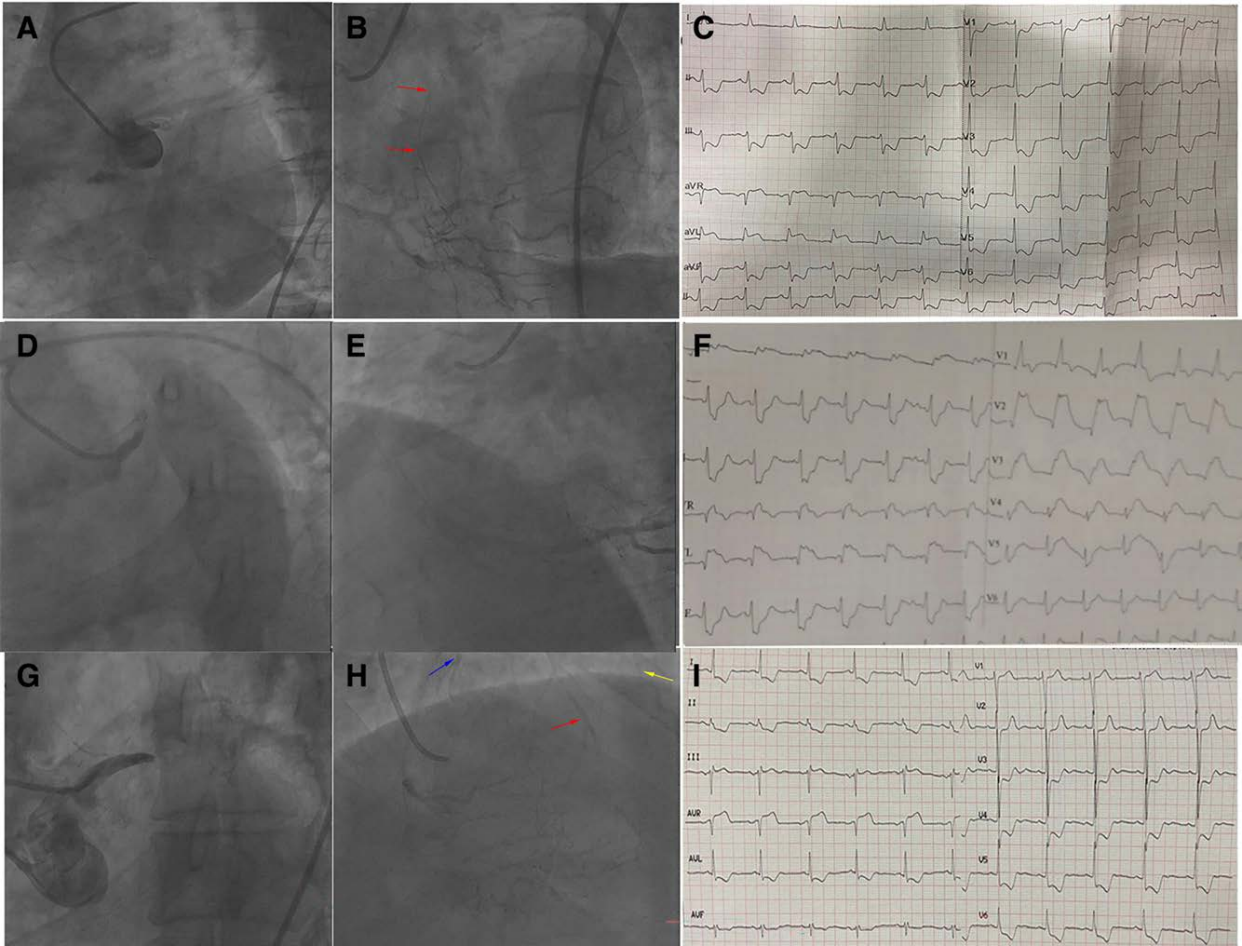
(A–C) illustrates STE in both aVR and aVL in a patient with LM occlusion and collateral filling of LAD (red arrow). (D–F) illustrates STEMI plus LAFB + RBBB in a patient with LM occlusion and no collateral circulation, and the patient died of cardiac shock. (G–I) illustrates STE in aVR in a patient with LM occlusion and collateral filling of the LAD (red arrow), diagonal branch (yellow arrow), and LCX (blue arrow). LAFB = left anterior fascicular block, LAD = left anterior descending coronary artery, LCX = left circumflex artery, LM = left main, STE = ST-segment elevation, STEMI = ST-segment elevation myocardial infarction, RBBB = right bundle branch block.

Collateral circulation (Rentrop score ≥ 1) from the RCA to LM was observed in 47 patients. The specificity of the STEMI pattern for collateral flow absence was 100%. Collateral filling of the LAD territory was observed in 62% (21/34) of patients with STE in aVR + aVL. In contrast, collateral filling of both LAD and LCX territories was observed in 73% (16/22) of patients with STE in aVR (Fig. 2B, *P* < .01). LAFB + RBBB predicted collateral circulation absence with a specificity of 87% and a sensitivity of 29% (*P* < .05). However, neither LAFB nor RBBB alone showed a significant association with collateral circulation. Patients without collateral circulation tended to have wider QRS intervals (122 ± 24 vs 110 ± 26, *P* < .05). Figure 4 illustrates 3 representative ECG types and the corresponding collateral filling territories in acute LM occlusion.

## 4. Discussion

The present study investigated the predictors of early mortality in patients with AMI caused by LM occlusion. To our knowledge, this present retrospective study provided the most extensive dataset on acute total LM occlusion. We first demonstrated that STEMI plus shock accurately predicted in-hospital mortality among these patients. Moreover, STEMI plus LAFB + RBBB predicted a fatal in-hospital outcome in LM occlusion with a high specificity.

A previous study reported that STE in aVR resulted in an adverse clinical outcome in STEMI patients,^[11]^ STE in aVR was associated with more in-hospital mortalities in a small sample of LM obstruction.^[12]^ In contrast, our present study indicated that STE in aVR was related to better survival in LM-AMI. This may be explained by well-developed collateral circulation alleviating myocardial ischemia in patients presenting with STE in aVR. Moreover, Iida et al^[13]^ described higher in-hospital mortality in patients with STE in lead aVL than STE in aVR in 89 cases of LM-AMI. However, our study demonstrated a lower mortality in patients presenting with STE in aVR + aVL. Both total occlusion and partial obstruction were enrolled in Takayuki study. The majority of the deceased were LM occlusion patients presenting with STE in aVR + aVL in their research. STE in aVR + aVL and STE in aVR predicted the presence of collateral circulation, while STEMI indicated the absence of collateral circulation. Accordingly, it was reasonable to conclude that STEMI predicted a higher mortality rate in LM occlusion. In patients with LM-AMI, the BCIS registry reported that STEMI was associated with an increase in the risk of 30-day mortality by 3-fold compared to NSTEMI.^[14]^ In the case of well-developed collateral circulation, the absence of STE in anterior and lateral leads may suggest a diagnosis of NSTEMI and delay the door-to-balloon time.

Cardiogenic shock is a common complication of acute total LM occlusion. A meta-analysis reported 56% of in-hospital mortality in 295 patients with LM-related AMI complicated by cardiogenic shock.^[1]^ Similarly, our present study demonstrated that a fatal in-hospital outcome was observed in 62% of patients presenting with cardiogenic shock. Well-developed collateral circulation was associated with a lower prevalence of cardiogenic shock and predicted survival in this clinical scenario. Hang Zhou et al^[15]^ reported a STEMI pattern in 25 patients with acute total LM occlusion without collateral flow; cardiogenic shock was present in 76% of all patients, and the in-hospital mortality rate was 88%. STE in anterior and lateral leads indicated the absence of collateral flow in the setting of LM occlusion. This ECG feature was associated with an increased cardiogenic shock risk. Thus, presentation with STEMI and cardiogenic shock simultaneously portended an extremely poor prognosis. Clinical physicians need to be highly alert to these patients, and emergency revascularization as early as possible may save the patients’ lives.

Bundle branch block at hospital admission in patients with AMI predicted in-hospital complications and poor short-term survival.^[16]^ The LAFB was more common than left posterior hemiblock and often accompanied by RBBB. Bifascicular block (RBBB + LAFB) complicating AMI was usually associated with the presence of heart failure and a poor prognosis.^[17]^ Because the proximal LAD supplies both the left anterior fascicular branch and right bundle branch simultaneously, the coexistence of LAFB and RBBB indicates a large area of jeopardized myocardium. Bifascicular block was associated with higher 1-year mortality than isolated RBBB in AMI.^[18]^ The prevalence of LAFB, RBBB, and LAFB + RBBB was 50 to 80, 9, and 17% in previous studies with a small patient series of LM occlusion, respectively,^[19–21]^ consistent with our findings. Bifascicular block was frequently seen in LM occlusion.^[22]^ Fiol et al^[23]^ first described 4 cases of STEMI plus LAFB + RBBB in 7 patients with acute LM occlusion without collateral flow; 3 patients with the above ECG feature died of cardiogenic shock. Several cases also reported STEMI plus LAFB + RBBB portended poor clinical outcomes in patients with total occlusive LM-AMI.^[24,25]^ Our previous study reported that STE in I, aVL, and V2 to V5 plus LAFB + RBBB was a specific predictor for acute total LM occlusion.^[7]^ In the present study, we further demonstrated that this ECG presentation was a valid diagnostic criterion and a feasible prognostic marker for LM occlusion. Clinicians should be on high alert for this ECG presentation in the emergency room, and prompt revascularization therapy may improve clinical outcomes.

The incidence of acute LM occlusion was 0.5% in AMI patients undergoing coronary angiography in our current study. This finding corroborated previous studies with an incidence range of 0.5% to 0.58%.^[4,26]^ The 5-year mace-free survival rate in the current study was 44%, consistent with previous reports.^[5,27]^ Despite the frequent use of advanced cardiac life support, early mortality remains extremely high within this STEMI subset.^[28]^ In several studies, neither IABP nor intravascular microaxial left ventricular assist device improved clinical benefit in patients with cardiogenic shock complicating AMI.^[29,30]^ Due to the small sample size, whether the use of advanced mechanical circulatory support (VA-ECMO, TandemHeart supporter, Impella® devices, or unloading VA-ECMO) in LM occlusion could improve clinical outcomes remains unclear.^[2,27,31]^ Further research is needed to evaluate the benefit of advanced mechanical circulatory support in the setting of LM occlusion. Overall, optimal management of patients with LM-AMI remains a significant challenge, especially for those with a high risk of early mortality (STEMI plus LAFB + RBBB or STEMI plus shock).

Study limitations: First, this was a small and retrospective cohort study, and intravascular microaxial left ventricular assist devices were unavailable in our center, so it was unclear whether the ECG predictors in our study were valid in patients with advanced hemodynamic support. Second, although we analyzed the annual mortality rate, the changes in medical protocols and management during the vast time range (27 years) may affect the exploration of predictors of mortality. Third, a selection bias resulting from sample exclusion and missing data cannot entirely be ruled out, because most patients with LM occlusion died before undergoing coronary angiography.

## 5. Conclusion

AMI due to acute total LM occlusion was associated with high in-hospital mortality. ECG features predicted in-hospital mortality and were associated with shock and collateral circulation. STEMI plus shock is a valuable predictor for in-hospital mortality in patients with acute total LM occlusion. STEMI plus LAFB + RBBB predicted a fatal in-hospital outcome in LM occlusion with high specificity. Prospective research is needed to further evaluate these predictors in LM occlusion.

## Author contributions

**Conceptualization:** Chunwei Liu.

**Methodology:** Fan Yang, Yuecheng Hu, Jingxia Zhang.

**Writing – original draft:** Chunwei Liu.

**Writing – review & editing:** Hongliang Cong, Ximing Li.
